# EAGLE: Explicit Alternative Genome Likelihood Evaluator

**DOI:** 10.1186/s12920-018-0342-1

**Published:** 2018-04-20

**Authors:** Tony Kuo, Martin C. Frith, Jun Sese, Paul Horton

**Affiliations:** 1 Artificial Intelligence Research Center, AIST, 2-3-26 Aomi, Koto-ku, Tokyo, 135-0064 Japan; 2 Department of Computational Biology and Medical Sciences, Graduate School of Frontier Sciences, The University of Tokyo, 5-1-5 Kashiwanoha, Kashiwa, Chiba, 277-8562 Japan; 3 AIST-Waseda CBBD-OIL, 3-4-1 Ookubo, Shinjuku-ku, Tokyo, 169-8555 Japan; 4 AIST-Tokyo Tech RWBC-OIL, 2-12-1 Okayama, Meguro-ku, Tokyo, 152-8550 Japan

**Keywords:** Next generation sequencing data analysis, Variant calling, Variant quality score, Genomic variants, Generative probabilistic models

## Abstract

**Background:**

Reliable detection of genome variations, especially insertions and deletions (indels), from single sample DNA sequencing data remains challenging, partially due to the inherent uncertainty involved in aligning sequencing reads to the reference genome. In practice a variety of ad hoc quality filtering methods are employed to produce more reliable lists of putative variants, but the resulting lists typically still include numerous false positives. Thus it would be desirable to be able to rigorously evaluate the degree to which each putative variant is supported by the data. Unfortunately, users who wish to do this, e.g. for the purpose of prioritizing validation experiments, have been faced with limited options.

**Results:**

Here we present EAGLE, a method for evaluating the degree to which sequencing data supports a given candidate genome variant. EAGLE incorporates candidate variants into explicit hypotheses about the individual’s genome, and then computes the probability of the observed data (the sequencing reads) under each hypothesis. In comparison with methods which rely heavily on a particular alignment of the reads to the reference genome, EAGLE readily accounts for uncertainties that may arise from multi-mapping or local misalignment and uses the entire length of each read. We compared the scores assigned by several well-known variant callers to EAGLE for the task of ranking true putative variants on both simulated data and real genome sequencing based benchmarks. For indels, EAGLE obtained marked improvement on simulated data and a whole genome sequencing benchmark, and modest but statistically significant improvement on an exome sequencing benchmark.

**Conclusions:**

EAGLE ranked true variants higher than the scores reported by the callers and can used to improve specificity in variant calling. EAGLE is freely available at https://github.com/tony-kuo/eagle.

**Electronic supplementary material:**

The online version of this article (10.1186/s12920-018-0342-1) contains supplementary material, which is available to authorized users.

## Background

Variant calling is an important task in genome analysis, and one with many remaining challenges. Previous studies have shown that different methods exhibit low concordance between their variant calls [1, 2] and the reproducibility of variant calling workflows has been thrown into question [3]. Insertion-deletions (indels) in particular pose many challenges [4, 5]. Yet, indel variants have an especially strong impact on phenotype [6] and disease [7, 8]. Thus, there is a strong need for accurate evaluation of putative indel variants. For example, a ranked list of putative variants can expedite experimental validation of variants.

Many well-known variant callers can evaluate the likelihood of a candidate variant given multiple samples in conjunction with population statistics or machine learning methods with fair accuracy [9–12]. However, these method often require large amounts of samples and/or population level data of known variants, such as dbSNP — a condition not met in many use cases. For example, a database of common variants may not be available for non-model organisms. Even for humans, mutations involved in rare inherited diseases may be unique to a patient or family. Thus, evaluating putative variants in a single-sample calling setting is an important endeavor in genomic research.

Here we present EAGLE, a method which explicitly evaluates how well sequencing data fit the alternative genome sequence implied by a putative variant using an explicit probability model that handles uncertainty in a well-principled manner. Our model uses read sequences in their entirety, thus requiring the flanking regions around the putative mutation to be supported by the data. In addition, we account for the uncertainty that is inherent in multi-mapped reads, ambiguous gap placements, and potentially misaligned reads from paralogs outside the reference genome. Earlier methods have previously considered multi-mapped reads for SNPs [13], however EAGLE is also applicable to indels and handles additional sources of uncertainty, such as local gap placement. Using both real and simulated benchmark data, we demonstrate that EAGLE is better at ranking putative variants than other scores and conclude that EAGLE can improve the specificity of variant callsets.

## Methods

### Generative probabilistic model of the read data

**Fig. 1:**
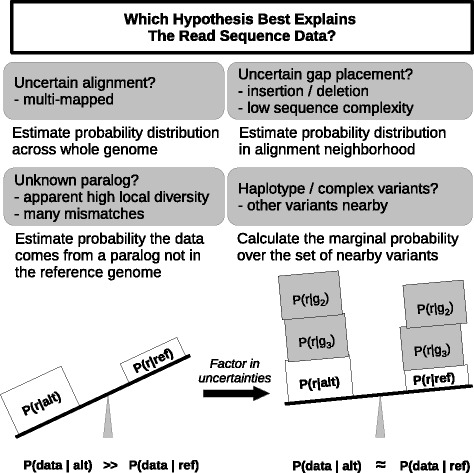
The model aims to handle the various uncertainties inherent in the variant calling process in order to calculate and compare the probability of the data given: the candidate variant sequence and the reference sequence. Simply, the more uncertain the data (*g*_*n*_ representing other possible sources of read *r*) the more uncertain the explanation

Our method is motivated by various uncertainties in the variant calling process, which we handle probabilistically (Fig. 1). At a high level, given sequencing data from a sample genome, one would like to have a way to compute which of two or more hypothetical sequences for the sample genome is more likely.

In our application at least three hypotheses are considered; one is that the sample genome is (homozygously) identical to the reference genome, and the other two are that the sample genome differs from the reference genome (homozygously or heterozygously respectively) to reflect a putative variant reported by some variant caller. For the heterozygous hypothesis, we assume that one allele is identical to the reference genome sequence and the other allele reflects the putative variant. Our method scores putative variants based on the likelihood each hypothesis given the data (more generally the posterior probability of the data given each hypothesis).

As detailed in the supplementary text (Additional file 1), our formulation makes some simplifying assumptions to reduce the problem to computing the probability of observing each read *r* given the hypothetical genome sequence *G* (reference or alternative). *G* is assumed to be diploid and is defined as a multiset of read length substrings, where substring *g* can be selected from either chromosome copy. We decompose this into the probability that the genome segment sequenced to produce *r* was *g* and the probability that (in the presence of sequencer error) sequencing *g* would produce read *r*.

$$\begin{aligned} P[\!r|G] &=\sum_{g\in G} P[\!g|G] P[\!r|g]\\ &\propto \sum_{g\in G} P[\!r|g]\qquad\quad\text{\scriptsize assuming uniform coverage}\\ &\propto \sum_{g \in G}{\prod_{i=1}^{\ell}{P[\!g_{i}|r_{i}]}}\quad\text{\scriptsize Bayes' Law with uniform priors}\\ P[\!g_{i}|r_{i}] &= \left\{\begin{array}{ll} 1 - e_{i}& \quad \text{if } r_{i} = g_{i} \\ \frac{e_{i}}{3} & \quad \text{otherwise} \end{array}\right. \end{aligned} $$

where *ℓ* is the length of read *r*, *g*_*i*_ is the *i*th base of *g* and *r*_*i*_ is the corresponding base in the read sequence with base-call error probability *e*_*i*_ (reflecting the quality score). In the derivation we assume uniform priors on the sequence genome segment *g* (i.e. uniform coverage) and a uniform prior on reads (of equal length, see Additional file 1). We also assume that there are no indel sequencing errors. Though the model could theoretically be extended to handle indel errors, the computational cost would be significant.

As written above, computing *P*[ *r*|*G*] entails summing over all of the length *ℓ* segments of the genome, which number ≈3×10^9^×2 strands×2 alleles for a diploid human genome; and this needs to be done for each read. Clearly this intractable, so in the interest of speed we invoke two approximations based on (multi)mapping of the reads onto the reference genome:

1. We assume reads which are not mappable to the neighborhood of (i.e. overlapping) the location of the candidate variant(s) will not affect the probability ratio of hypotheses, and can therefore be ignored.
2. When summing the probability of the remaining reads, we only consider genome segments overlapping the location(s) where each read maps. Notably, the genome segments considered for each read may differ.

Unfortunately, the initial step of this approximation scheme suffers from reference bias. That caveat notwithstanding, by summing over all locations a read maps to; and for each of those locations, summing over all overlapping segments; we account for the main uncertainties that arise in pileup-based variant calling methods — multi-mapping and ambiguous gap placement.

### Mini-haplotype hypotheses

Genome variants can occur together in the genome, often within the span of a single read. Thus, we should consider *clusters* of putative variants which occur within a threshold distance of another putative variant. We conceptually chain those together and explicitly consider combinatorial genome hypotheses representing as many possible subsets of those putative variants together as computational resources allow. In the default parameter settings used for this study, EAGLE chains putative variants within 10 bp together and tries up to 1024 combinations of those putative variants; i.e. for up to ten neighboring variants, all possible combinations are tried.

For simplicity we do not consider combinatorial hypotheses of mixed zygosity, in which some putative variants are heterozygous and others are homozygous. Nevertheless in some cases we do consider a large number of hypotheses, which suggests that something akin to multiple hypotheses testing correction might be appropriate. To address this we adjust the prior probability of combinatorial hypotheses — always giving the reference sequence a prior of 50% and dividing the remaining 50% evenly among the alternative hypotheses for any given cluster. Note this “prior” reflects the strength of our belief in the variant genome hypothesis before EAGLE examines the read data but *after* knowing that this candidate variant caller was listed as a putative variant by the variant caller used. Thus it is distinct from an estimation of the overall frequency of genetic polymorphisms in the population.

The underlying EAGLE probabilistic model computes the likelihood of individual genome hypotheses, treating all hypotheses in a uniform way. EAGLE has an option to output these raw likelihoods for users interested in individual hypotheses which may include multiple nearby putative variants. To rank individual putative variants, EAGLE combines these likelihoods (weighted by their priors) in the form of a marginal posterior probability of the data given the variant. Summarizing the above in mathematical notation yields:

$$\frac{P[R|v]}{P[R|\text{not }v]} = \frac{\sum_{G \in \mathbf{G_{v}}}{P[G] \, P[R|G]}}{\sum_{G \in \mathbf{G_{u}}}{P[G] \, P[R|G]}}\\ $$

$$P[G]= \left\{\begin{array}{cl} 0.5 &\text{if}\ G=G_{\text{ref}}\\ \frac{0.5}{|\mathbf{G_{v}}| + |\mathbf{G_{u}}| - 1}& \text{otherwise} \end{array}\right. $$

where *R* is the read data, *G*_*v*_ is the set of hypotheses containing the putative variant *v* (in general combined with other nearby putative variants), and *G*_*u*_ is the set of hypotheses not including *v* but possibly including nearby putative variants.

### Outside source of reads

The human genome is repetitive; paralogs and low complexity regions often approximately match many other locations in the genome. The possibility that a read mappable to a given genome segment actually derives from a paralog recorded in the reference genome is naturally covered by our formulation as described above. However, the sequenced sample genome may have some additional *outside paralogs* (e.g. extra copies of Alu or other repetitive elements) which are similar enough to copies in the reference genome to make reads derived from them mappable even though their true origin does not directly correspond to any position in the reference genome. Genome sequencing data is expected to contain many such *reference-external* reads, albeit less so for exome sequencing.

Reference-external reads pose a serious risk of generating false positive putative variants, because they may appear in multiple read alignment pileups at genome positions they are not derived from. Filtering out reads based on minimum mapping quality score can alleviate this risk [14] but no threshold can perfectly distinguish reference-external reads from other reads. Just as reference-external reads may mislead pileup-based variant calling, they might also mislead the probabilistic model of EAGLE if all reads were forced to be explained as originating from inside the reference framework. Without an outside source of reads, the likelihood ratio would be unduly influenced by reference-external reads which map somewhat poorly (i.e. with more mismatches than expected from their quality scores), to a genome segment containing a putative variant; but even more poorly to the reference sequence (or vice versa) — therefore misleadingly appearing to support one hypothesis over another, when in fact they support neither.

Considering the issues discussed above, we defined an integrated model of inside and outside sources of reads as follows:

$$\begin{aligned} \frac{P[\!r|G_{v}]}{P[\!r|G_{u}]} &= \frac{P[\!r \mathrm{~from~paralog}] \; + \; \sum_{g \in \substack{\mathrm{variant~segments}\\ \mathrm{containing~{v}}}} P[\!r|g]} {P[\!r \mathrm{~from~paralog}] \; + \; \sum_{g \in \substack{\text{corresponding}\\ \mathrm{reference~segments}}} P[\!r|g]}\\ {} P[\!r \mathrm{~from~paralog}] &= P[\!r \mathrm{ \ from~inside~paralog}] \!+ P[\!r \mathrm{~from ~outside ~paralog}]\\ & = \sum_{g \in \substack{\text{paralogous} \\ \text{neighborhoods}}} P[\!r|g] \;\; + \;\; m_{r} h {\sum_{\substack{f \colon \\ \text{HD}\left(f,r^*\right) \leq 1}}  \prod_{i=1}^{\ell_r} P\left[\!f_i|r_{i}\right]} \end{aligned} $$

where *m*_*r*_ is the number of places read *r* maps to in the reference genome; *r*^∗^ is the sequence called by the read (i.e. the sequence given by the read assuming no errors); HD(*f*,*r*^∗^) denotes the Hamming distance between *f* and *r*^∗^; and *h* is a parameter of EAGLE. All results reported here use a value of *h*=10^−4^, a value we derived empirically based on preliminary simulation studies. The outside paralog term contains a multiplicative factor of *m*_*r*_ to model our expectation that genome segments with many paralogs in the inside genome are more likely to have paralogs outside of the reference framework as well. Thus, the outside paralog term serves to down-weight false evidence based on reference-external reads. In the supplementary text (Additional file 1) we provide a derivation of the outside paralog term as an approximation of the probability of *r* being generated under a probabilistic source of outside paralogs. However, if read length does not vary, a constant outside paralog term may suffice (Additional file 1: Figure S1).

### EAGLE software

**Fig. 2:**
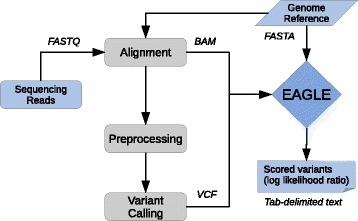
A high level overview of the EAGLE workflow. EAGLE requires: read alignments in BAM format, a set of candidate variants in VCF format, and the reference genome as a FASTA file. Preprocessing in this study refers to preprocessing steps described in GATK best practices

We implemented the ideas above as a C program named EAGLE, available at https://github.com/tony-kuo/eagle. EAGLE uses standard file formats and can easily be added to existing variant calling pipelines. Figure 2 gives an overview of the workflow we used for this paper.

## Results

In this section we describe two tests of EAGLE’s performance; one using simulated reads generated from an *in silico* alternative human genome (based on the NS12911 genome), and one using real reads from the NA12878 benchmark dataset.

### NS12911 variants with simulated reads

We conducted a simulation study where we know the entire ground truth by reconstructing the diploid sequence of chromosome 22 of an individual (the NS12911 human genome), using a list of phased variants against the human reference genome (hg19) provided by a Sanger sequencing based assessment [15]. We then simulated paired-end reads of length 100 bp, with insert size 500±30 bp at ∼30× coverage, using DNemulator [16].

The variants from NS12911 capture the challenge of evaluating putative variants in mutational hotspots and in low complexity and other repetitive regions. Notably, real indels often occur in repetitive regions [6] which introduces more uncertainty in calling and makes evaluation more difficult.

For this benchmark, we mapped reads to the hg19 reference genome using BWA MEM [17]; and performed duplicate sequence removal, indel realignment, and base recalibration according to the pre-processing steps from GATK ‘best practices’ [14] (see Additional file 1 for details). We used the resulting BAM format alignment data to call variants with: GATK HaplotypeCaller (3.3.0) [9, 18], SAMtools mpileup (1.3) [19], FreeBayes (1.0.2) [11], and Platypus (0.8.1) [12]. Each callset was normalized using vt normalize [20] and the vcfallelicprimitives module in vcflib (https://github.com/ekg/vcflib) to deconstruct complex variants.

From the known NS12911 variants, we determined the number of true positive (TP) and false positive (FP) calls (Additional file 1: Table S1). For simplicity of evaluation, here and in the other benchmarks, we considered a called variant correct if it matches the gold standard in sequence and position, regardless of zygosity.

**Fig. 3:**
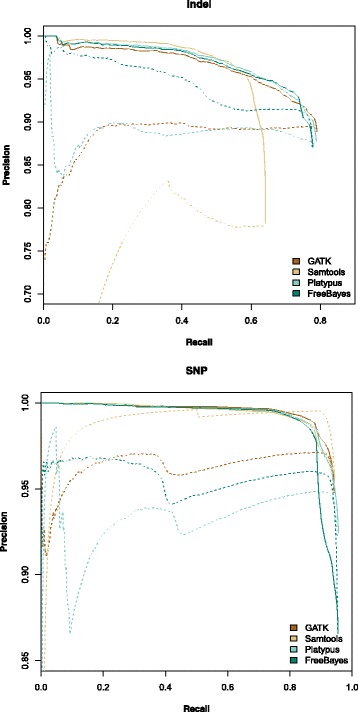
Precision vs Recall of NS12911 based ∼30× fold coverage simulated reads is shown for Indels (top) and SNPs (bottom). Solid lines represent EAGLE’s likelihood. Dotted lines represent the caller’s quality score. Recall levels are shown in increments of 50 variant calls with the maximum level based on the number of variants in the NS12911 benchmark set. The variant calls were ranked based on our model’s marginal posterior probability or each caller’s quality score respectively. Precision is the fraction of high ranking variants which are correct, plotted over a wide range of thresholds

We calculated the marginal posterior probabilities for variants in each callset and compared them to each caller’s quality scores for their ability to evaluate candidate variants (Fig. 3). The results show a dramatic improvement in precision when using our model to rank both indels and SNPs. We obtained consistent results when we reduced the sequencing coverage to ∼10× fold (Additional file 1: Figure S2); EAGLE varied only slightly from ∼30× coverage results, the variant callers scores changed more but the increase in precision gained by using EAGLE remained.

Since we only simulated reads from chromosome 22, all variant calls located on other chromosomes must have been due to spurious read mappings. For GATK indels, only 14 out of 174 variant calls at other chromosomes had likelihood ratios that favored the alternative hypothesis, with a top rank of 5856. Similarly for SNPs, only 159 out of 1584 variant calls had likelihood ratios that favor the alternative hypothesis, with a top rank of 21913. In comparison to variants called in chromosome 22, 8792 out of 9436 indels and 35031 out of 37794 SNPs had likelihood ratios that favored the alternative hypothesis. These results show that our formulation for outside paralogs is effective.

Although we observed that some indel false positives were highly ranked, manual examination of these variants revealed that these calls were in the correct position but not completely correct in sequence or length due to repetitive sequences. In these cases, the called variant is often still better supported than the reference genome hypothesis. It is up for debate whether these variants should be considered false positives or whether positional correctness is sufficient. However, inferring the effect of a mutation (e.g. amino acid substitution, frameshift, etc.) generally requires the exact mutation sequence; and in this study we required calls to be correct in sequence as well as position.

As can be observed at the high recall levels in Fig. 3, our model ranked some true SNPs very low. We examined these cases and observed that almost all of them are regions of high diversity where some variants are spaced just far enough apart that we did not combine them in mini-haplotype hypotheses. Thus it may be beneficial to explore using a larger distance threshold, albeit at the cost of longer computation time.

### NA12878 benchmark variant calls

We tested our model on real sequencing data from NA12878 (cell line of an individual from a CEPH pedigree), using an exome sequencing dataset (Garvan HG001) from Genome-In-A-Bottle (GIAB) [21] and a 200× whole genome sequencing dataset from Illumina Platinum Genomes (IPG). The benchmark from IPG consists of a high confidence callset for the GRCh38 human reference genome constructed using FreeBayes, Platypus, and GATK variant callers. The benchmark from GIAB consists of a high confidence callset for hg19 which was constructed using FreeBayes, Samtools, and GATK using a number of sequencing libraries from different sequencing technologies, which were then integrated. Because the benchmarks were constructed from variant calls made by the same tools we are comparing against, there may be some bias in the following results.

We performed variant calling, normalization, and calculated variant probabilities as described above for the GIAB exome sequencing data and the IPG whole genome sequencing data separately (Additional file 1: Table S2), The IPG workflow was conducted on the GRCh38 human reference genome. The EAGLE computation time non-negligible, but generally requires less time than the variant calling step used to generate candidate variants (Additional file 1: Table S3).

**Fig. 4:**
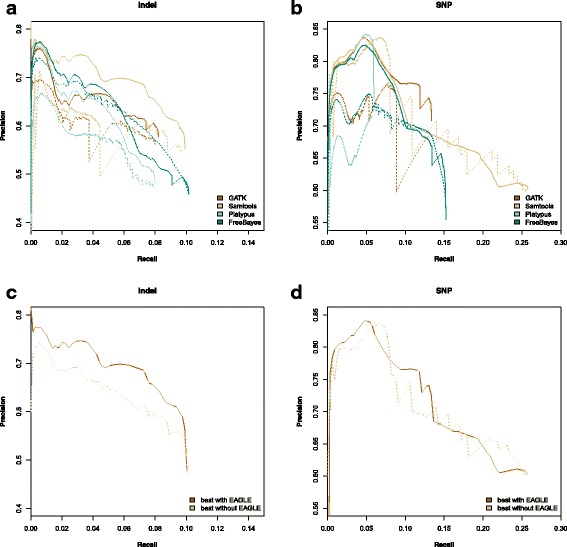
Precision vs Recall for NA12878 using benchmarks from exome sequencing GIAB Indels (**a**, **c**) and SNPs (**b**,**d**). Plots **a**) and **b**) show the full precision vs recall for each method. Solid lines represent EAGLE’s likelihood. Dotted lines represent the caller’s quality score. Recall levels are shown in increments of 50 variant calls with the maximum level based on the number of variants in the GIAB benchmark set. The variant calls were ranked based on our model’s marginal posterior probability or each caller’s quality score respectively. Precision is the fraction of high ranking variants which are correct, plotted over a range of thresholds. Plots **c**) and **d**) show the best precision at a given recall among all methods with EAGLE versus among all methods without EAGLE, for indels and SNPs respectively

**Fig. 5:**
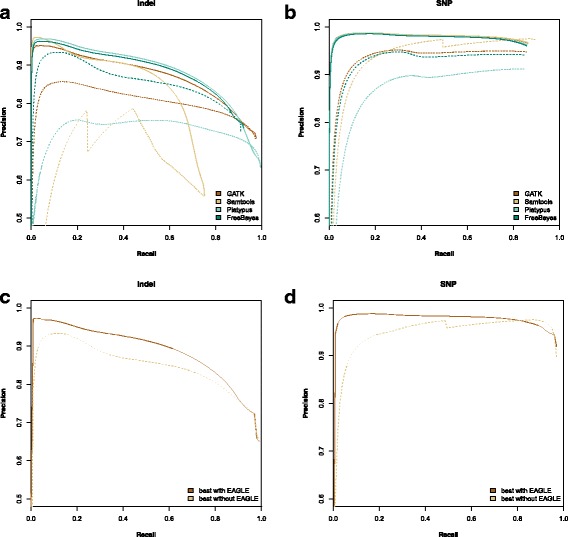
Precision vs Recall for NA12878 using benchmarks from whole genome sequencing Illumina Platinum Genome Indels (**a**, **c**) and SNPs (**b**,**d**). Plots **a**) and **b**) show the precision vs recall for each method. Solid lines represent EAGLE’s likelihood. Dotted lines represent the caller’s quality score. Recall levels are shown in increments of 50 variant calls with the maximum level based on the number of variants in the GIAB benchmark set. The variant calls were ranked based on our model’s marginal posterior probability or each caller’s quality score respectively. Precision is the fraction of high ranking variants which are correct, plotted over a wide range of thresholds. Plots **c**) and **d**) show the best precision at a given recall among all methods with EAGLE versus among all methods without EAGLE, for indels and SNPs respectively

We evaluated our model using precision versus recall as described above and evaluated EAGLE for GIAB (Fig. 4) and IPG (Fig. 5). The IPG results show that EAGLE generally has better precision for both SNPs and indels, especially at low recall.

In general, there is a statistically significant improvement in the ranking of variants when using the EAGLE likelihood over the caller’s native scoring system (for all callers, the p-value is less than the R language Mann-Whitney-Wilcoxon test reporting limit of 10^−16^). We also tested the Variant Quality Score Recalibration (VQSR) method (see Additional file 1), though due to technical limitations, we were only able to apply it to the GATK callset for the NA12878 benchmark.

We examined the set of false negatives in the GIAB tests (variants in the benchmark given low probability by our model) and observed that low read depth accounted for the majority. As we only utilized one exome sequencing dataset, compared to the many libraries used to generate the benchmark, the low sensitivity overall and uncertainty in low read coverage regions is reasonable for both the callers and our model. This is corroborated by the much higher precision seen in the IPG tests, where the results and degree of improvement obtained by using our model was more similar to what we observed with simulated reads. We also compared EAGLE’s likelihood ratio with VQSR on the GATK callset (Additional file 1: Figure S3) and observed that EAGLE has better performance for all but IPG SNPs, even though VQSR requires a large external training dataset to function.

Examining the set of false positives, especially at low recall, we saw that almost all putative variants had abnormally high read depth (> 1000 for indels, > 10000 for SNPs) which affected all callers, as well as EAGLE, and likely indicates copy number variations. Indeed, the top 10 false positive GIAB SNPs (in terms of likelihood ratio) had very high read depths and are all copy number variations listed in the Database of Genomic Variants [22].

Finally, we note that the issue of alternative representations of equivalent complex variants complicated the analysis. In the standard variant calling format (VCF) one entry describes a single SNP, insertion, or deletion event with only one nucleotide used as the context sequence. Thus complex variants such as ACACCACCACC to AA must be split into at least two VCF entries, and unfortunately different variant callers sometimes differ in how they do this (Additional file 1: Table S4).

## Discussion

As described above we measured the performance of EAGLE using both real and simulated sources of benchmark variants, each with their strengths and weaknesses. For real data, the absolute ground truth is not available, so we followed the typical practice of comparing against a conservative benchmark of high confidence calls that are considered to be a subset of all true variants in a genome. Ranking putative variants with EAGLE consistently improved precision compared to ranking by the callers’ variant quality scores on both exome (Fig. 4) and whole genome sequencing (Fig. 5); and in particular for the GIAB exome dataset indels, the Samtools callset ranked by EAGLE yielded a marked improvement in precision over a wide range of recall values (Fig. 4b). We note that some of the PR curves are unusual in the sense that precision of the best ranking putative variants (i.e. the far left-hand side of the PR curve) is relatively *low*, going against the usual expectation of an approximately monotonic transition from high to low precision as the acceptance cut-off is lowered to increase recall. This may be partially explained by a limitation of this type of real data benchmark, namely the fact that variants not in the benchmark set are always treated as false positives, even though some of them may be true. Notably, since by definition such “false false positives” are the true variants overlooked by the variant callers used to construct the benchmark data, treating them as false positives may systematically bias performance evaluation in favor of those variant callers.

Simulated read data generated from a known genome has the advantage that we know the absolute ground truth. On the other hand, simulated reads are not a perfect model of real sequencing data as the simulation software cannot fully account for the various sources of noise and systematic error which exist in practice. In any case, on simulated data, as in real data, ranking by EAGLE also consistently improved variant calling precision (Fig. 3).

What enables EAGLE to improve the precision of variant calling vis-à-vis the variant quality score of the callers? Conceptually, EAGLE is nearly unique in its use of explicit alternative hypotheses and its computation of genotype likelihoods in a manner which is independent of the details of the pileup, in contrast with the base pileup model employed by nearly all variant callers. Of course many concepts employed by EAGLE are not completely novel. Numerous previous methods apply probabilistic reasoning to variant calling [9–13, 23] and some methods also perform haplotype inference to improve accuracy [11, 12]. Principled methods to handle reads likely to derive from paralogs have been described as well [13, 23].

Practically speaking, to the best of our knowledge there is no tool available which evaluates candidate variants in the manner that EAGLE does. The closest analog is VQSR, a machine learning method acting on variant calling summary statistics and is not broadly applicable to non-model organisms. In summary, although defined by a relatively simple, explicit model, EAGLE combines many advantages of previous methods to effectively address the uncertainties depicted in Fig. 1.

On the other hand, many variant calling methods offer features that we have not explored for EAGLE. For example, variant calling from multiple samples [9–11], and supervised learning based post-processing to improve accuracy by incorporating additional information such as strand bias and unusual read depth [14, 24]. In principle this approach can improve performance when a sufficient number of known variants is available for training. Although EAGLE performed competitively in the limited comparison we were able to make between it and VQSR, one future direction would be to try combining EAGLE with machine learning techniques to further improve performance. Another direction we are considering is extending the probabilistic model to better handle copy number variations, as our results indicate room for improvement in that area. Finally we note that cancer related somatic variant calling is typically performed in a single sample framework and calling of short indels has been shown to be difficult [25]. We speculate that it may be possible to beneficially integrate the posterior probabilities computed by EAGLE into procedures for somatic variant calling.

## Conclusion

We developed EAGLE, a method for evaluating candidate genome variants based on an explicit, probabilistic generative model of read data given a hypothetical genome sequence. Using both simulation and real benchmark data, we compared EAGLE with several well-known variant callers and demonstrate that our model is able to rank putative variants better than current methods, leading to marked improvement in precision at comparable recall levels.

## Additional file

Additional file 1 We provide one additional file holding supplementary text. The document includes: a detailed mathematical derivation of the EAGLE probabilistic model outlined here, empirical evaluation of the effect of varying the outside paralog term (**Fig. S1**), a measurement of performance with reduced sequence coverage (**Fig. S2**), additional performance comparison between EAGLE and VQSR (**Fig. S3**), summary statistics of variant calls made by various callers on NS12911 (**Table S1**) and on GIAB and IPG benchmarks (**Table S2**), running time of EAGLE on some representative tasks (**Table S3**), an example variant which GATK, Platypus and FreeBayes represent with distinct VCF output (**Table S4**); and a description of a simple mutation planting based evaluation of EAGLE and the results (**Fig. S4**, **Table S5**). (PDF 1945 kb)

## Abbreviations

BAM: Binary alignment map
BWA MEM: Burrows-wheeler aligner memory
CEPH: Centre d’
Etude du Polymorphism Humain; DNA: DeoxyriboNucleic acid
dbSNP: The single nucleotide polymorphism database
EAGLE: Explicit alternative genome likelihood evaluator
GATK: Genome analysis toolkit
GIAB: Genome-in-a-bottle
GRCh38: Genome reference consortium human build 38
IPG: Illumina platinum genomes
PR: Precision-recall
SNPs: Single nucleotide polymorphisms
VCF: Variant call format
VQSR: Variant quality score recalibration
