# Supplementary material for: EAGLE: Explicit Alternative Genome Likelihood Evaluator
Source: BMC Med Genomics. 2018 Apr 20;11(Suppl 2):28. doi: 10.1186/s12920-018-0342-1 (PMC5918433; doi:10.1186/s12920-018-0342-1)
Supplement: Supplementary file 1 — We provide one additional file holding supplementary text. The document includes: a detailed mathematical derivation of the EAGLE probabilistic model outlined here, empirical evaluation of the effect of varying the outside paralog term (Fig. S1), a measurement of performance with reduced sequence coverage (Fig. S2), additional performance comparison between EAGLE and VQSR (Fig. S3), summary statistics of variant calls made by various callers on NS12911 (Table S1) and on GIAB and IPG benchmarks (Table S2), running time of EAGLE on some representative tasks (Table S3), an example variant which GATK, Platypus and FreeBayes represent with distinct VCF output (Table S4); and a description of a simple mutation planting based evaluation of EAGLE and the results (Fig. S4, Table S5). (PDF 1945 kb) [file 12920_2018_342_MOESM1_ESM.pdf]

EAGLE: Explicit Alternative Genome Likelihood Evaluator Supplemental Text

Tony Kuo[1], Martin C. Frith[1], Jun Sese[1] & Paul Horton[1*]

S1 Mathematical Model

S1.1 Overview

We derive a probability distribution of the observed data (the sequencing reads) conditioned on an entire hypothetical genome (reference or alternative) and then describe a simple but effective way to use traditional read mapping to approximate the computation. Our model is conceptually close to a previous model by Simola & Kim [1], but the details of our method and the mathematical exposition we present here differ substantially from their work.

Throughout this paper we assume that sequencers do not make indel errors. This is not essential to our theoretical approach, but simplifies the derivation and greatly reduces computation time. The results we obtained suggest this assumption is tenable for the Illumina sequencing platform.

S1.2 Basic Model

To describe our model, we start by deriving the likelihood of one sequencing read r of length ℓ_r, whose base-call quality scores define a probability distribution over some length ℓ_s DNA sequence s:

$$P[s|r] = \prod_{i=1}^{\ell_r} P[s_i|r_i]. \tag{1}$$

where s_i is the ith base of s and r_i is the probability vector over $\{\texttt{a,c,g,t}\}$ corresponding to the base and quality score of the ith position of r. It is useful to reverse the direction of the conditional with Bayes' law producing:

$$P[r|s] = \frac{P[r]}{P[s]} \prod_{i=1}^{\ell_r} P[s_i|r_i]$$

where $P[s]$ is the prior probability of a DNA sequence s when we know only that s was read by a sequencer. For simplicity, we assume no indel errors so that the length of each read is the same as the length of the segment it derives from. The sequencing technology and experimental protocol employed will determine the prior probability distribution of the length of reads (and equivalently the length of segments read) which we denote as $\mathcal{L}(\ell)$ (by definition $\sum_\ell \mathcal{L}(\ell) \equiv 1$). Furthermore, we assume that the prior probability of equal length sequence segments are equal. Thus,

$$P[s] = \frac{\mathcal{L}(\ell_s)}{4^{\ell_s}}$$

[1]Artificial Intelligence Research Center, AIST, Japan
[*]Corresponding Author

where 4 is the alphabet size of possible nucleotides. A similar type of distribution could be assumed for $P[r]$ using a larger alphabet size to include quality score information, but for our purposes it is more useful to leave $P[r]$ unspecified, as it will cancel later on when we look at probability ratios. Thus,

$$P[r|s] = \frac{P[r]}{P[s]} \prod_{i=1}^{\ell_r} P[s_i|r_i] = \frac{P[r] 4^{\ell_s}}{\mathcal{L}(\ell_s)} \prod_{i=1}^{\ell_s} P[s_i|r_i]$$

is the probability of a read given that we know it derives from a length ℓ_r DNA sequence s. But what if we only know that it derives from somewhere in genome G? Then,

$$P[r|G] = \sum_{g \in G} P[g|G] P[r|g]$$

where $P[g|G]$ is the probability that genome sequence segment g is sequenced, given we know only that some segment from G is sequenced. Although often unrealistic, we assume uniform priors on genome segments g of any particular length (i.e. uniform coverage) and further assume that the length distribution of g follows the general prior defined above as $\mathcal{L}(\ell)$. Using n_{ℓ_g} to denote the number of genome segments of length ℓ_g:

$$P[g|G] = \mathcal{L}(\ell_g) \frac{1}{n_{\ell_g}}$$

and combining,

$$\begin{aligned} P[r|G] = \sum_{g \in G} P[g|G] P[r|g] &= \sum_{g \in G} \left(\mathcal{L}(\ell_g) \frac{1}{n_{\ell_g}} \right) \frac{P[r] 4^{\ell_g}}{\mathcal{L}(\ell_g)} \prod_{i=1}^{\ell_g} P[g_i|r_i] \\ &= \sum_{g \in G} \frac{P[r] 4^{\ell_g}}{n_{\ell_g}} \prod_{i=1}^{\ell_g} P[g_i|r_i] \\ &= \sum_{g \in G} \frac{P[r]}{n_{\ell_g}} \prod_{i=1}^{\ell_g} 4P[g_i|r_i] \\ &\approx \frac{P[r]}{n} \sum_{g \in G} \prod_{i=1}^{\ell_g} 4P[g_i|r_i] \end{aligned}$$

where the approximation in the last line reflects the fact that since chromosomes are typically much longer than reads, the number of genome segments n_{ℓ_g} can be well approximated as the genome length n. Later, we will explicitly consider genome hypotheses of differing length, but as the length of these differences will be small compared to the genome length, we will still use n to approximate the number of segments of any given length for all genome hypothesis.

One important detail is that in practice some fraction ξ (mnemonic for *xenos*) of the reads will derive from sequences *outside* of the reference sequence coordinates. Such reads may derive from sequences unrelated to the human genome (e.g. contamination) or regions of the human genome not fully covered in the reference (e.g. telomere regions). Later we will revisit how to model the outside source of reads, but first we proceed with a generic outside source which simply assumes

that the reads from the outside follow the same distribution as the general prior $P[r]$.

$$\begin{aligned} P[r|H] = &\ P[\text{from outside}]\, P[r|\text{from outside}] \\ &+ P[\text{from G}]\ P[r|\text{from G}] \\ &= \xi P[r] + (1-\xi)\frac{P[r]}{n}\sum_{g\in G}\prod_{i=1}^{\ell_g} 4P[g_i|r_i] \end{aligned}$$

To compute the probability of the entire set of reads we assume that each read is generated independently, an assumption which is not true considering biases introduced by PCR amplification and other steps of the sequencing process. Nonetheless we make this assumption of read independence, to obtain:

$$\begin{aligned} P[R|H] &= \prod_{r\in R}\left(\xi P[r] + (1-\xi)\frac{P[r]}{n}\sum_{g\in G}\prod_{i=1}^{\ell_g} 4P[g_i|r_i]\right) \\ &= \prod_{r\in R} P[r]\left(\xi + (1-\xi)\frac{1}{n}\sum_{g\in G}\prod_{i=1}^{\ell_g} 4P[g_i|r_i]\right) \end{aligned}$$

Equivalently, by Bayes' law

$$P[H|R] = \frac{P[H]}{P[R]}\prod_{r\in R} P[r]\left(\xi + (1-\xi)\frac{1}{n}\sum_{g\in G}\prod_{i=1}^{\ell_g} 4P[g_i|r_i]\right)$$

The ratio of the probability of two alternative hypotheses H_v and H_u, assuming genome sequences G_v and G_u respectively is:

$$\begin{aligned} \frac{P[H_v|R]}{P[H_u|R]} &= \frac{\frac{P[H_v]}{P[R]}\prod_{r\in R} P[r]\left(\xi + (1-\xi)\frac{1}{n}\sum_{g\in G_v}\prod_{i=1}^{\ell_g} 4P[g_i|r_i]\right)}{\frac{P[H_u]}{P[R]}\prod_{r\in R} P[r]\left(\xi + (1-\xi)\frac{1}{n}\sum_{g\in G_u}\prod_{i=1}^{\ell_g} 4P[g_i|r_i]\right)} \\ &= \frac{P[H_v]\prod_{r\in R}\left(\xi + (1-\xi)\frac{1}{n}\sum_{g\in G_v}\prod_{i=1}^{\ell_g} 4P[g_i|r_i]\right)}{P[H_u]\prod_{r\in R}\left(\xi + (1-\xi)\frac{1}{n}\sum_{g\in G_u}\prod_{i=1}^{\ell_g} 4P[g_i|r_i]\right)} \end{aligned}$$

This equation can be slightly cleaned up by defining convenience constants $x = n\xi$ and $h = 1-\xi$, yielding:

$$\begin{aligned} \frac{P[H_v|R]}{P[H_u|R]} &= \frac{P[H_v]\prod_{r\in R}\left(\xi + (1-\xi)\frac{1}{n}\sum_{g\in G_v}\prod_{i=1}^{\ell_g} 4P[g_i|r_i]\right)}{P[H_u]\prod_{r\in R}\left(\xi + (1-\xi)\frac{1}{n}\sum_{g\in G_u}\prod_{i=1}^{\ell_g} 4P[g_i|r_i]\right)} \\ &= \frac{P[H_v]\prod_{r\in R}\left(\frac{x}{n} + h\frac{1}{n}\sum_{g\in G_v}\prod_{i=1}^{\ell_g} 4P[g_i|r_i]\right)}{P[H_u]\prod_{r\in R}\left(\frac{x}{n} + h\frac{1}{n}\sum_{g\in G_u}\prod_{i=1}^{\ell_g} 4P[g_i|r_i]\right)} \\ &= \frac{P[H_v]\prod_{r\in R}\left(x + h\sum_{g\in G_v}\prod_{i=1}^{\ell_g} 4P[g_i|r_i]\right)}{P[H_u]\prod_{r\in R}\left(x + h\sum_{g\in G_u}\prod_{i=1}^{\ell_g} 4P[g_i|r_i]\right)} \end{aligned}$$

where G is treated as the multiset of all length ℓ segments of the hypothetical genome (including both chromosomes in each pair and for whole genome sequencing both strands, while for exome sequencing the targeted regions of the genome).

As written, this computation iterates over each genome position *once for each read*, requiring an unrealistic amount of computation time. Fortunately, we can overcome this problem. First, we note that that the probability $P[r|g]$ will be negligible for the vast majority of genome segments g. Only segments similar to the consensus sequence of r will contribute significantly to the sum — precisely the segments which read mapping software, used in multimap mode, are designed to find quickly.

Some more notation is needed to explain our approximation precisely. Consider the likelihood ratio of two genome hypothesis, e.g. the reference genome G_u (u mnemonic for unchanged) and a variant genome G_v obtained by slightly editing G_u in accordance to a candidate genome variant, say by deleting one base, at position e of the reference genome. Further let us denote the set of reads which cover position e when mapped to the reference genome as R^e and the set of genome segments to which read $r \in R^e$ maps to in the reference or variant genome as G_u^r and G_v^r respectively. Importantly, although by definition the reads in R^e all map covering position e of the reference, in general they may map to multiple regions of the genome, and those regions may differ between different reads in R^e. Finally we define the *neighborhood* N_u^r (N_v^r) as the set of length ℓ_r genome segments overlapping any segment in G_u^r or G_v^r respectively. By summing the likelihood over these neighborhoods instead of just a single segment, we cover the case in which the exact alignment of a read to the genome (i.e. gap placement) is ambiguous — as is often the case for indel variants found in repetitive regions and/or occurring simultaneously with other nearby variants. This summation is necessary because in the case of ambiguous local alignments more than one local length ℓ genome segment could generate read r with non-negligible probability.

Now we can express our approximation of the likelihood ratio as:

$$\frac{P[H_v|R]}{P[H_u|R]} = \frac{P[H_v] \prod_{r \in R_e} \left(x + h \sum_{g \in N_v^r} \prod_{i=1}^{\ell_g} 4P[g_i|r_i] \right)}{P[H_u] \prod_{r \in R_e} \left(x + h \sum_{g \in N_u^r} \prod_{i=1}^{\ell_g} 4P[g_i|r_i] \right)}$$

Note that although we make an effort to treat the reference and variant genome hypothesis symmetrically, in the formulation as stated some asymmetry remains. Namely, we define the read set R^e relevant to a candidate variant in terms of which reads map near position e in the reference genome. Ideally, the set of reads considered would be the union of those which map near position e in either the reference genome *or the variant genome*, which (especially in the case of longish indels) might not be the same as R^e. Defining R^e only in terms of the reference genome is a practical decision which eases program implementation — all of the reads can be mapped to the reference genome in a precomputation step and those results used to quickly determine R^e for each candidate variant (which number in the millions). To remove this reference bias, one would need a way to quickly query the read set for reads similar to a genome segment overlapping the edited region of each variant genome hypothesis. In principle a suffix array or similar type of index built from the reads could be used to do this, but we leave that for future work. Nevertheless, although the set of reads considered potentially relevant to a given candidate variant is biased towards the reference genome, the rest of the computation is completely symmetric, explicitly handling genome segments unique to the alternative genome and the probability mass of each $r \in R^e$ derived from sites distant from e.

S1.3 Outside Source Revisited

The generic outside source of reads described in our derivation above is inadequate for both technical and biological reasons. In technical terms, the only reads for which a generic source may have a non-negligible posterior probability are reads which are dissimilar to any region of the hypothetical (reference or alternative) genome, i.e. unmappable reads, but in practice we ignore such reads. If our implementation could be improved to perform read mapping to both hypotheses, the generic outside source could play a role in preventing the probability of the read in the hypothesis to which it does not map from becoming excessively small — but in our current implementation we only map to the reference genome. In terms of biology, there is a need to model paralogous sequences found in the individual genome but not in the reference genome; for example an individual might have an extra occurrence of the Alu repetitive element distinct from any of the numerous Alu elements included in the reference genome. If the extra copy is a recent one, its sequence may be close enough to its paralogous brethren to allow for reads deriving from it to map to the reference genome. Thus our method will see those reads, and potentially make incorrect inferences about candidate variants based on them if this clearly *non-generic* outside source of reads is not modeled effectively.

S1.3.1 Outside Paralog Source

Here we describe a semi-theoretical model of an outside source of sequences paralogous to the human genome. We admit up front that some of the assumptions will seem forced. In fact, this model is reverse engineered from practice. Nevertheless we feel it is useful to put what we are doing in an explicit theoretical framework in the hope that exposing the remaining issues will facilitate future improvement of the method and/or theory by ourselves or others. Our generative model of outside paralogs assumes every genome segment g, potentially has an additional copy in the sequenced individual which is absent in the reference genome. We use F to denote the "shadow" of G and f^g to denote the doppelgänger paralog of g in F (`f` is mnemonic for "far", cognate to "para", and `F` is an alphabetic neighbor of `G`). Armed with that notation we seek to model the following:

$$P[r|F] \;=\; \sum_{g \in G} \Big(\; P[g|F] \; \sum_{f} P[f|g] \, P[r|f] \; \Big)$$

Where $P[g|F]$ is the probability that a read originating from the outside paralog source is derived from the doppelgänger copy of g. $P[f|g]$ is the probability of a particular sequence f given that it is an outside paralog derived from a copy of segment g of the inside genome G, and $P[r|f]$ is the probability of a read given sequence f (more generally written as $P[r|s]$). Realistically modeling $P[g|F]$ would require consideration of the differing tendencies for various types of sequences to repeat frequently, for example known repetitive elements are much more likely to be repeated polymorphically than segments which occur uniquely in the reference genome. However for simplicity we choose to assume a uniform distribution over the starting position of g and the general prior distribution $\mathcal{L}(\ell_r)$ on the length of any read derived from a copy of g, so $P[g|F] = \frac{\mathcal{L}(\ell_r)}{n}$. Turning to $P[f|g]$, a realistic model would require assumptions about the distribution of age and mutation rate of paralogs which we do not want to deal with. Instead we start with the less ambitious observation that since by assumption f is a paralogous copy of g, f should be similar to g, and $P[f|g]$ should be negligible unless f is similar to g. The least informative probability distribution consistent with that assumption is to assign an equal probability to all sequences similar to g. This begs the question of how *similar* is to be defined; which for practical reasons we define in a procedural way via mappability. More precisely, we only consider the possibility that a read r might come from

the doppelgänger copy of g if r (multi)maps to g when aligned to G. This is roughly equivalent to viewing the mapping sensitivity threshold as a lower bound on the degree of dissimilarity for which $P[f|g]$ becomes small enough to ignore.

$$\begin{aligned}
P[r|F] &= \sum_{g\in G}\Big(\, P[g|F] \;\sum_{f} P[f|g]\, P[r|f] \,\Big) \\
&\approx \sum_{g\colon r \text{ maps to } g}\Big(\, P[g|F] \sum_{\substack{f \text{ similar} \\ \text{to } r}} \frac{1}{\nu_{\ell_r}}\, P[r|f] \,\Big) \\
&= \sum_{g\colon r \text{ maps to } g}\Big(\, \frac{\mathcal{L}(\ell_r)}{n} \sum_{\substack{f \text{ similar} \\ \text{to } r}} \frac{1}{\nu_{\ell_r}}\; P[r|f] \,\Big) \\
&= \frac{m_r}{n\nu_{\ell_r}} \sum_{\substack{f \text{ similar} \\ \text{to } r}} \mathcal{L}(\ell_r)\, P[r|f]
\end{aligned}$$

Where m_r is the number of segments in G to which r maps; and ν_{ℓ_r} is the size of the set "f similar to r", subscripted with ℓ_r to indicate that the size of this set grows with ℓ_r. The set "f similar to r" is not precisely defined, but could be considered to be implicitly defined by the condition that g and r are similar enough that r is mappable to g. This follows from the assumption that r is a read obtained by sequencing f (a paralog of g). In any case, in practice we replace the set "f similar to r" with a precisely defined one in an approximation given below.

As before we define the prior probability of a DNA sequence, given only that we know it was sequenced to produce some read r, as $P[f] = \frac{\mathcal{L}(\ell_r)}{4^{\ell_r}}$; and apply Bayes' law to obtain a formula in which quality scores can be used, obtaining:

$$\begin{aligned}
\sum_{f} \mathcal{L}(\ell_r) P[r|f] &= \sum_{\substack{f \text{ similar} \\ \text{to } r}} \mathcal{L}(\ell_r) \frac{P[r]P[f|r]}{P[f]} \\
&= P[r] \sum_{\substack{f \text{ similar} \\ \text{to } r}} \mathcal{L}(\ell_r) \frac{\prod_{i=1}^{\ell_r} P[f_i|r_i]}{\frac{\mathcal{L}(\ell_r)}{4^{\ell_r}}} \\
&= P[r] \sum_{\substack{f \text{ similar} \\ \text{to } r}} \prod_{i=1}^{\ell_r} 4P[f_i|r_i]
\end{aligned}$$

Now we make another approximation.

$$\begin{aligned}
\sum_{\substack{f \text{ similar} \\ \text{to } r}} \prod_{i=1}^{\ell_r} P[f_i|r_i] \;&\approx\; a \sum_{\substack{f\colon \\ \mathrm{HD}(f,r^*)\leq 1}} \prod_{i=1}^{\ell_r} P[f_i|r_i] \\
&\coloneqq a\, \mathcal{S}_{\mathrm{H}_1(r)} \qquad \text{(defined for subsequent notational convenience)}
\end{aligned}$$

Where r^* is the sequence called by the read (i.e. the sequence given by the read assuming no errors), $\mathrm{HD}(f, r^*)$ denotes the Hamming distance between f and r^*, and a is a proportionality constant. In other words the assumption is that summing over sequences with zero or one mismatches relative to the read is an acceptable approximation to summing over all sequences similar to r. Note that although not completely obvious from the formula above, $\mathcal{S}_{\mathrm{H}_1(r)}$ can be computed in time proportional to ℓ_r by taking advantage of the fact that changing one character in f only changes

one term in $\sum[f_i|r_i]$. Although motivated by computation cost, the approximation may be a reasonably good one in practice. For example consider a read of length 100 with all quality scores corresponding to a 1% error rate. The probability the read r was sequenced from its consensus sequence would be $0.99^{100} \approx 0.366$, while the probability it was sequenced from one with exactly one substitution would be $100*0.01*0.99^{99} = 0.99^{98} \approx 0.370$. So in this case, the Hamming distance ≤ 1 set of sequences together account for over 73% of the probability mass. In the hope of slightly improving the approximation we multiply by a constant $a \geq 1$, making the somewhat weaker assumption that the sum over the strings within Hamming distance one will be approximately *proportional* (as opposed to equal) to the full probability.

We can now replace the generic outside source of reads defined in the previous section to obtain:

$$\begin{aligned} P[r|H] = \; & P[\text{from outside}]\, P[r|\text{from outside}] \\ & + P[\text{from G}]\; P[r|\text{from G}] \\ & = \xi P[r] m_r \frac{a}{n\nu_{\ell_r}} 4^{\ell_g}\, \mathcal{S}_{\mathrm{H}_1(r)} + (1-\xi)\frac{P[r]}{n} \sum_{g\in G}\prod_{i=1}^{\ell_g} 4P[g_i|r_i] \end{aligned}$$

Assuming the reads are generated independently:

$$\begin{aligned} P[R|H] &= \prod_{r\in R}\left(\xi P[r] m_r \frac{a}{n\nu_{\ell_r}} 4^{\ell_g}\, \mathcal{S}_{\mathrm{H}_1(r)} + (1-\xi)\frac{P[r]}{n} \sum_{g\in G}\prod_{i=1}^{\ell_r} 4P[g_i|r_i]\right) \\ &= \frac{1}{n}\prod_{r\in R} P[r]\left(\xi m_r \frac{a}{\nu_{\ell_r}} 4^{\ell_g}\, \mathcal{S}_{\mathrm{H}_1(r)} + (1-\xi) \sum_{g\in G}\prod_{i=1}^{\ell_r} 4P[g_i|r_i]\right) \end{aligned}$$

Applying Bayes' law,

$$\begin{aligned} \frac{P[H_v|R]}{P[H_u|R]} &= \frac{P[H_v]}{P[H_u]} \frac{\prod_{r\in R}\left(\xi m_r \frac{a}{\nu_{\ell_r}} 4^{\ell_g}\, \mathcal{S}_{\mathrm{H}_1(r)} + (1-\xi)\sum_{g\in G_v}\prod_{i=1}^{\ell_r} 4P[g_i|r_i]\right)}{\prod_{r\in R}\left(\xi m_r \frac{a}{\nu_{\ell_r}} 4^{\ell_g}\, \mathcal{S}_{\mathrm{H}_1(r)} + (1-\xi)\sum_{g\in G_u}\prod_{i=1}^{\ell_r} 4P[g_i|r_i]\right)} \\ &= \frac{P[H_v]}{P[H_u]} \frac{\prod_{r\in R}\left(\xi m_r \frac{a}{\nu_{\ell_r}}\, \mathcal{S}_{\mathrm{H}_1(r)} + (1-\xi)\sum_{g\in G_v}\prod_{i=1}^{\ell_r} P[g_i|r_i]\right)}{\prod_{r\in R}\left(\xi m_r \frac{a}{\nu_{\ell_r}}\, \mathcal{S}_{\mathrm{H}_1(r)} + (1-\xi)\sum_{g\in G_u}\prod_{i=1}^{\ell_r} P[g_i|r_i]\right)} \\ &\approx \frac{P[H_v]}{P[H_u]} \frac{\prod_{r\in R}\left(\xi m_r \frac{a}{\nu_{\ell_r}}\, \mathcal{S}_{\mathrm{H}_1(r)} + (1-\xi)\sum_{g\in N_v^r}\prod_{i=1}^{\ell_r} P[g_i|r_i]\right)}{\prod_{r\in R}\left(\xi m_r \frac{a}{\nu_{\ell_r}}\, \mathcal{S}_{\mathrm{H}_1(r)} + (1-\xi)\sum_{g\in N_u^r}\prod_{i=1}^{\ell_r} P[g_i|r_i]\right)} \\ &= \frac{P[H_v]}{P[H_u]} \frac{\prod_{r\in R}\left(\frac{\xi}{1-\xi} m_r \frac{a}{\nu_{\ell_r}}\, \mathcal{S}_{\mathrm{H}_1(r)} + \frac{1-\xi}{1-\xi}\sum_{g\in N_v^r}\prod_{i=1}^{\ell_r} P[g_i|r_i]\right)}{\prod_{r\in R}\left(\frac{\xi}{1-\xi} m_r \frac{a}{\nu_{\ell_r}}\, \mathcal{S}_{\mathrm{H}_1(r)} + \frac{1-\xi}{1-\xi}\sum_{g\in N_u^r}\prod_{i=1}^{\ell_r} P[g_i|r_i]\right)} \\ &= \frac{P[H_v]}{P[H_u]} \frac{\prod_{r\in R}\left(m_r h\, \mathcal{S}_{\mathrm{H}_1(r)} + \sum_{g\in N_v^r}\prod_{i=1}^{\ell_r} P[g_i|r_i]\right)}{\prod_{r\in R}\left(m_r h\, \mathcal{S}_{\mathrm{H}_1(r)} + \sum_{g\in N_u^r}\prod_{i=1}^{\ell_r} P[g_i|r_i]\right)} \end{aligned}$$

Where in the last line, the convenience constant h is redefined as $h = \frac{\xi a}{(1-\xi)\nu_{\ell_r}}$. In practice h is a tunable parameter of Eagle and it is not necessary to decompose it into its constituent components ξ, ν_{ℓ_r} and a. The theory described above suggests that the value of h should decrease with the read length of r, but currently Eagle uses the same value of h for all reads.

Down-weighting multi-mapped reads: We note in passing that with the paralog outside source model, multi-mapped reads which potentially support a candidate variant are weighted down in two ways. First, before even looking at the details of the read, the simple fact that it maps to so many places *inside* the reference genome, suggests it may be more likely to also map *outside* the reference genome if those extra sequences were available; an effect modeled by the m_r term. Second, even if we assume the read comes from the inside, its probability mass will be distributed among the multiple sites it maps to in accordance with the details (sequence and quality scores) of the read.

S1.3.2 Effectiveness of Outside Paralog Model in Practice

To investigate the practical impact of the outside paralog model, we compared the performance of candidate variant ranking using EAGLE with the full model outside term versus using EAGLE with a constant outside paralog term. We used the average value of the full model outside term over all candidate variants for the value of the constant. As shown in Figure S1, the impact on performance of using the full outside model was small and not even consistently beneficial. This anti-climatic result suggests that for the benchmarks we used, it is sufficient to have an appropriately weighted regularizer term to dampen the likelihood ratio in cases for which all hypotheses considered are poorly supported by the reads. However, we would note that the full outside model is designed to be robust in the face of putative variants found in repetitive regions — the hardest case for variant calling. We optimistically speculate that such difficult to call variants are under-represented in the gold standard set of variants benchmarked here, and the full outside model might demonstrate its value if variants in repetitive regions were more fully catalogued. Also, although the datasets we handled have uniform length reads, we note that the performance of a constant outside term would be expected to degrade for variable read-length datasets. The theoretical reasoning for this expectation is that the inside probability decreases rapidly with increased read length, so to maintain balance the outside term should also decrease with read length.

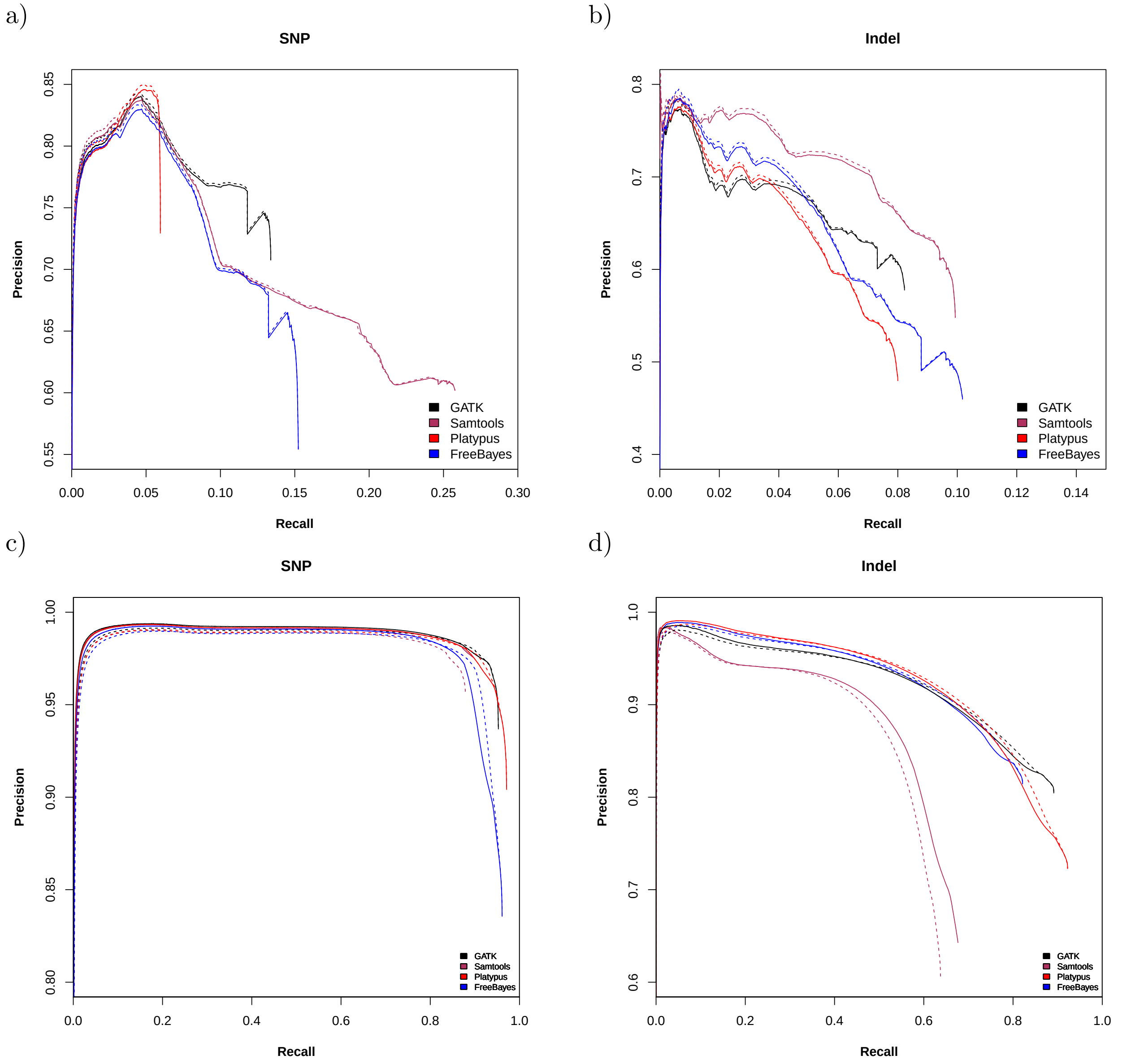

Figure S1: Exact versus constant outside paralog term performance. Precision versus recall on NA12878 cell line exome sequencing SNPs (a) and indels (b), and whole genome sequencing SNPs (c) and indels (d) are shown. Performance was scored using the GIAB and Illumina Platninum Genome for exome and whole genome sequencing respectively. Solid lines represent the exact paralog term. Dotted lines represent the constant paralog term. The average value of the exact outside paralog term was used for the value of the constant.

a) b)

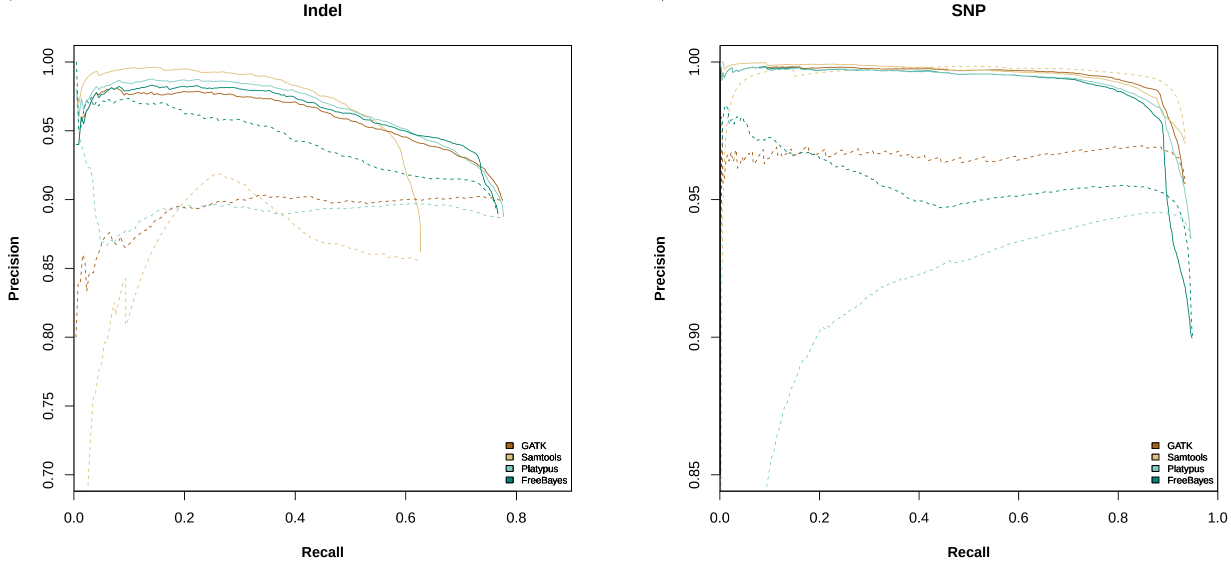

Figure S2: Precision vs Recall of NS12911 based ∼10× fold coverage simulated reads is shown for indels (a) and SNPs (b). Solid lines represent EAGLE's likelihood. Dotted lines represent the caller's quality score. Recall levels are shown in increments of 50 variant calls with the maximum level based on the number of variants in the NS12911 benchmark set. The variant calls were ranked based on our model's marginal posterior probability or each caller's quality score respectively. Precision is the fraction of high ranking variants which are correct, plotted over a wide range of thresholds.

S2 Supplemental Results

S2.1 Benchmarking Procedure

To simulate reads from the NS12911 genome, first we applied the phased variants to hg19 to obtain the heterozygous genome. Then we used dnemulator to simulate reads from chromosome 22 using the command:

- fasta-paired-chunks -f 500 -s 30 -l 100 -n 13000000 chr22.SIM.true.fa chr22.SIM.R1.fa chr22.SIM.R2.fa

The fastq quality scores were modeled using a real exome sequencing dataset, DRX000842 from the myelodysplasia study described in the first simulation benchmark using the command:

- fastq-sim chr22.SIM.R1.fa MDS-04N.R1.fq > chr22.SIM.R1.fq
- fastq-sim chr22.SIM.R2.fa MDS-04N.R2.fq > chr22.SIM.R2.fq

We then performed variant calling after mapping the simulated reads to the original hg19 reference genome.

For variant calling, we followed pre-processing steps described in GATK 'best practices' to construct a BAM file ready for variant calling as follows:

1. Read mapping with BWA-MEM
2. Label read groups with Picard AddOrReplaceReadGroups
3. Remove duplicate reads with Picard MarkDuplicates
4. Realign indels with GATK RealignerTargetCreator and IndelRealigner
5. Base quality recalibration with GATK BaseRecalibrator (with dbsnp 142) and PrintReads

We then performed variant calling for each caller as follows:

- GATK: HaplotypeCaller -R REF.fa -I F.recal_reads.bam -dontUseSoftClippedBases -stand_call_conf 20.0 -stand_emit_conf 20.0 -o F.gatkraw.vcf
- Samtools: mpileup -u -v -E -C 50 -t SP -f REF.fa F.recal_reads.bam | bcftools call -vc - > F.mpileup.vcf
- Platypus: callVariants –bamFiles=F.recal_reads.bam –refFile=REF.fa –output=F.platypus.vcf
- FreeBayes: freebayes –fasta-reference REF.fa F.recal_reads.bam > F.freebayes.vcf

After variant calling, we also attempted to use the machine learning method, Variant Quality Score Recalibration (VQSR), to score the callsets of each variant caller. However, we were only able to analyze the GATK results with VQSR, despite trying various combinations of training data and annotation sets. For Samtools and FreeBayes, the VariantRecalibrator tool exited with an error before finishing. On the Platypus callset it was able to finish, but not all variants in the callset were annotated with VQSR scores. In our simulation experiment using planted NS12911 mutations, we only simulated reads from chr22, which does not overlap with enough variants in the training data to allow training VQSR.

S2.2 Comparison of EAGLE and VQSR

We compared the performance of EAGLE and VQSR for NA12878 exome and whole genome sequencing datasets (Figure S3). We ran VQSR on the GATK variant calls for the NA12878 exome sequencing and WGS benchmarks with training sets: hapmap 3.3, 1000G omni 2.5, dbsnp (v138 for exome, v1 for WGS), and Mills 1000G gold standard indels; obtained from the GATK resource bundles along with the corresponding 'best practice' recommended parameters.

Tentatively, the performance is similar except for WGS indels and exome sequencing SNPs at mid-recall where EAGLE has significantly better precision. However, one advantage to VQSR is its ability to identify variants with abnormally high read depths (indicating CNVs or perhaps PCR errors), which EAGLE does not account for currently (as seen in low recall levels for WGS SNPs). We also note that the number of called variants suitable for VQSR training on this benchmark dataset is probably unusually high. Although the VQSR documentation recommends 30 exome samples to obtain the "thousands" of training points needed, a single NA12878 exome sequencing yields $\sim$350,000 training points. This likely does not represent the typical use case.

S2.3 Simple performance evaluation using a doctored reference genome

The section describes the results of an additional performance evaluation not mentioned in the main text. We conducted a simple simulation study using real human exome sequencing data from a myelodysplasia study [2] normal sample (DRX000842); but using a doctored version of the reference genome with planted "mutations". This scheme provides a partial gold standard for variant calling, for example if we add a 6bp insertion into the doctored reference genome, a good variant caller should detect that discrepancy as a 6bp deletion in the sample genome. This simulation scheme is rather limited, but it does have the benefit of using real sequencing reads, and can therefore give some indication of whether our assumption of read independence is tenable.

First, we randomly planted a total of 300 fake mutations into the exons of the GRCh37p13 human reference genome: 100 SNPs, 100 deletions, and 100 insertions each, with indel length up to 30 bp. We then mapped reads to the doctored reference genome using BWA MEM [3]; and performed duplicate sequence removal, indel realignment, and base recalibration according to the pre-processing steps from GATK 'best practices' [4] (see supplementary for details). We used the resulting BAM format alignment data to call variants with: GATK HaplotypeCaller (3.3.0) [5, 6], SAMtools mpileup (1.3) [7], FreeBayes (1.0.2) [8], and Platypus (0.8.1) [9]. Each callset was normalized using `vt normalize` [10] and the `vcfallelicprimitives` module in vcflib (https://github.com/ekg/vcflib) to deconstruct complex variants.

For each variant caller's callset, we evaluated: the planted mutations, called variants that neighbor the planted mutations, and 1000 randomly selected called SNPs and indels respectively. We then compared our likelihood ratio to the caller's quality scores in its ability to rank variants. Note that while many of these randomly selected calls may be correct, some probably are not; while we know with certainty that the planted mutations are correct. Thus all things being equal, a ranking which ranks the planted mutations higher is plausibly of higher quality. Overall, the EAGLE results were encouraging (Table S5, Figure S4).

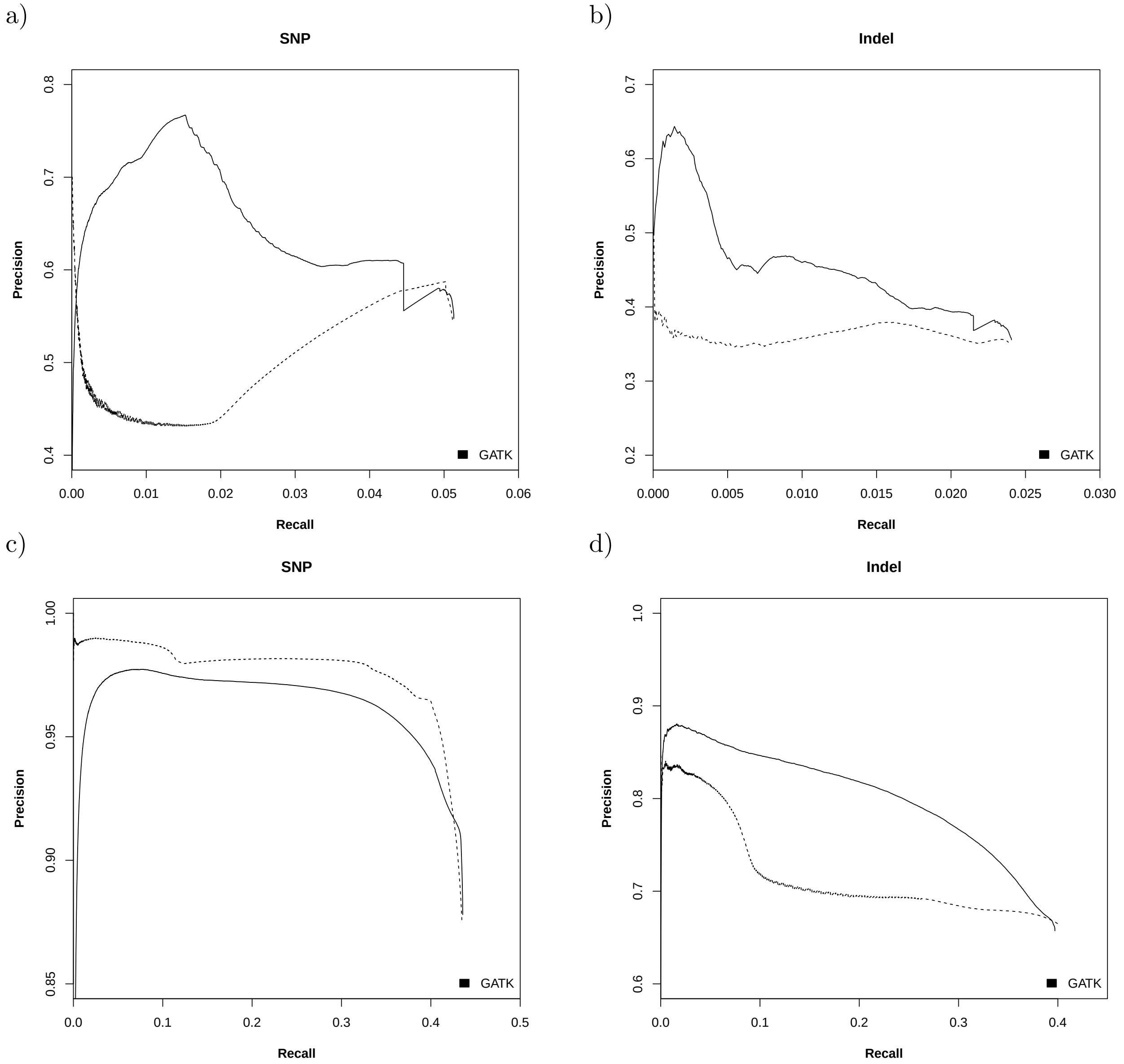

Figure S3: Performance of EAGLE versus VQSR. Precision and recall on NA12878 cell line exome sequencing SNPs (a) and indels (b), and whole genome sequencing SNPs (c) and indels (d) are shown. Performance was scored using the GIAB and Illumina Platninum Genome for exome and whole genome sequencing respectively. Solid lines represent EAGLE's likelihood. Dotted lines represent the VQSR score on the GATK callset, following the parameters recommended by 'best practices'. The results shown exclude the variant calls used in VQSR training (∼50% of the callset).

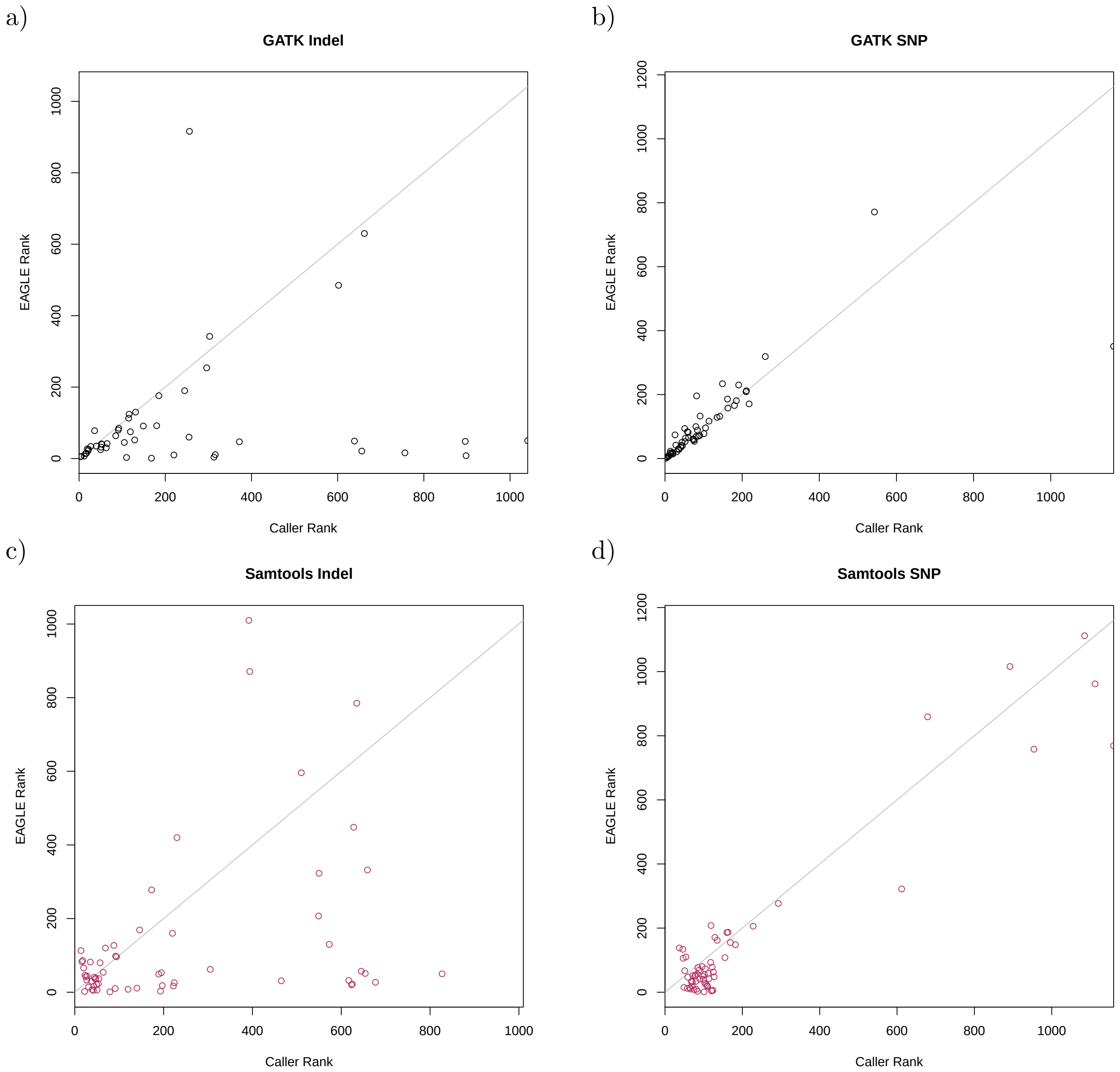

a)
GATK Indel
EAGLE Rank
Caller Rank
b)
GATK SNP
EAGLE Rank
Caller Rank
c)
Samtools Indel
EAGLE Rank
Caller Rank
d)
Samtools SNP
EAGLE Rank
Caller Rank

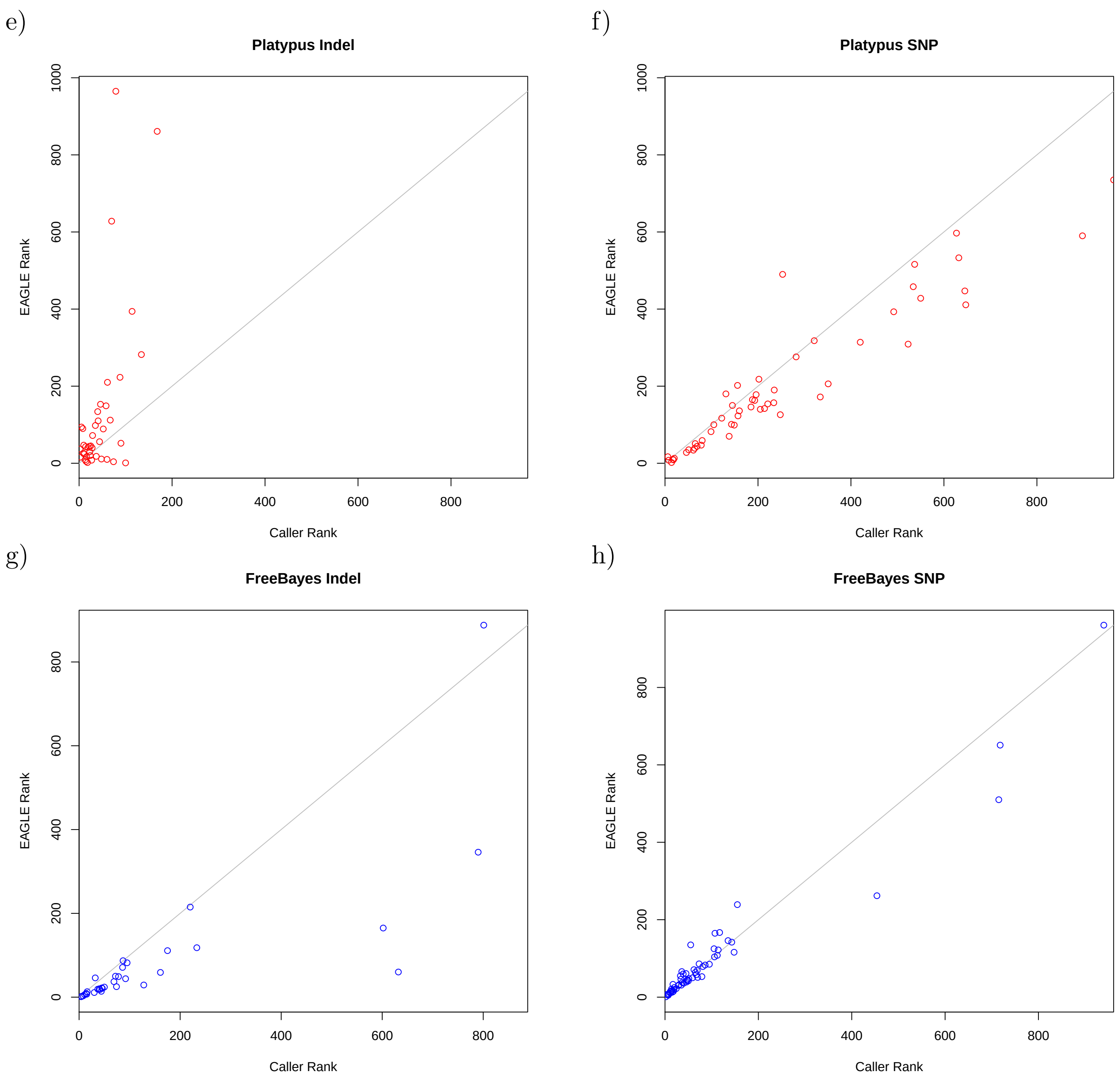

Figure S4: Variant caller ranks (x-axis) versus our model's ranks (y-axis) for planted mutations showing for each variant caller, indels and SNPs. Points in the lower-right indicate that the variant caller ranked a planted variant lower than our model and vice versa for the upper-left.

Supplemental Tables

Table S1: Variant calls from various tools with true positives (TP) and false positives (FP) with respect to NS12911.

	SNP: TP	FP	*Indel*: TP	FP
NS12911	39611	—	10794	—
GATK	36721	2015	8299	658
SAMtools	36806	1575	6845	1822
Platypus	37062	3077	8282	817
FreeBayes	37202	5624	8279	1016

Table S2: The number of correct SNP, correct indel and total variant calls for four callers is shown for the GIAB and IPG benchmarks.

	true SNP	true Indel	Total Calls
GIAB (truth)	2740734	174997	—
GATK	435972	42595	705886
Samtools	839087	51347	1495574
Platypus	193952	41426	358370
FreeBayes	496373	52699	1044665

	true SNP	true Indel	Total Calls
IPG (truth)	3785616	712607	—
GATK	3489337	559334	4683103
Samtools	3471006	430674	4483808
Platypus	3508630	571402	4965316
FreeBayes	3498677	528425	5153419

Table S3: NA12878 Runtime (wall-clock, hrs) using 8 cores (2.53 GHz). VC represents the time required for the variant calling procedure excluding read mapping. Pre-processing (mark duplicates, indel realignment, base quality score recalibration) required 5.78 hrs and 94.07 hrs for GIAB and IPG respectively, and is included in the VC time (note that some variant callers and some steps in pre-processing are not multi-processing capable). EAGLE represents the time required to run EAGLE on each callset. *Immediately before publication of this manuscript (after the times reported in this table were measured) we implemented computational optimizations which make the current version of EAGLE run faster (to be described elsewhere).*

	GIAB exome			IPG WGS		
	Num. Variants	VC	EAGLE	Num. Variants	VC	EAGLE
GATK	705289	18.68	4.11	4683103	151.17	91.04
SAMtools	1495574	14.03	5.19	4483808	155.92	63.72
Platypus	358370	6.06	2.34	4965316	100.72	85.95
FreeBayes	1044665	8.88	6.11	5153419	124.51	77.35

Table S4: Equivalent variants can be represented in multiple distinct ways.

Caller	Chrom.	Position	Original	Alternative
GATK	chr1	55541338	AC	A
	chr1	55541340	ACCACCACC	A
Platypus	chr1	55541338	ACACCACCAC	A
	chr1	55541348	C	A
FreeBayes	chr1	55541338	ACACC	A
	chr1	55541343	ACCACC	A

Table S5: Median ranks of mutations planted in a doctored reference genome, as described in section S2.3.

SNP :	EAGLE	Caller	*Indel*: EAGLE	Caller
GATK	**69**	72	**42**	116
SAMtools	**57**	103	**46**	94
Platypus	**150**	188	45	**35**
FreeBayes	51	**48**	**29**	69

References

[1] Simola,D.F. and Kim,J. (2011) Sniper: improved SNP discovery by multiply mapping deep sequenced reads. *Genome Biology,* **12**, R55.

[2] Yoshida,K., Sanada,M., Shiraishi,Y., Nowak,D., Nagata,Y., Yamamoto,R., Sato,Y., Sato-Otsubo,A., Kon,A., Nagasaki,M., Chalkidis,G., Suzuki,Y., Shiosaka,M., Kawahata,R., Yamaguchi,T., Otsu,M., Obara,N., Sakata-Yanagimoto,M., Ishiyama,K., Mori,H., Nolte,F., Hofmann,W.K., Miyawaki,S., Sugano,S., Haferlach,C., Koeffler,H.P., Shih,L.Y., Haferlach,T., Chiba,S., Nakauchi,H., Miyano,S., and Ogawa,S. (2011) Frequent pathway mutations of splicing machinery in myelodysplasia. *Nature,* **478**(7367), 64–69.

[3] Li,H. (2013) Aligning sequence reads, clone sequences and assembly contigs with BWA-MEM. arXiv:1303.3997v2 [q-bio.GN].

[4] Van der Auwera,G.A., Carneiro,M.O., Hartl,C., Poplin,R., del Angel,G., Levy-Moonshine,A., Jordan,T., Shakir,K., Roazen,D., Thibault,J., Banks,E., Garimella,K.V., Altshuler,D., Gabriel,S., and DePristo,M.A. (2013) From FastQ Data to High-Confidence Variant Calls: The Genome Analysis Toolkit Best Practices Pipeline. *Current Protocols in Bioinformatics,* **11**(1110), 11.10.1–33.

[5] McKenna,A., Hanna,M., Banks,E., Sivachenko,A., Cibulskis,K., Kernytsky,A., Garimella,K., Altshuler,D., Gabriel,S., Daly,M., and DePristo,M.A. (2010) The Genome Analysis Toolkit: A MapReduce framework for analyzing next-generation DNA sequencing data. *Genome Research,* **20**(9), 1297–1303.

[6] DePristo,M.A., Banks,E., Poplin,R., Garimella,K.V., Maguire,J.R., Hartl,C., Philippakis,A.A., del Angel,G., Rivas,M.A., Hanna,M., McKenna,A., Fennell,T.J., Kernytsky,A.M., Sivachenko,A.Y., Cibulskis,K., Gabriel,S.B., Altshuler,D., and Daly,M.J. (2011) A framework for variation discovery and genotyping using next-generation DNA sequencing data. *Nature Genetics,* **43**(5), 491–498.

[7] Li,H., Handsaker,B., Wysoker,A., Fennell,T., Ruan,J., Homer,H., Marth,G., Abecasis,G., Durbin,R., and Subgroup,.G.P.D.P. (2009) The Sequence Alignment/Map format and SAMtools. *Bioinformatics,* **25**(16), 2078–2079.

[8] Garrison,E. and Marth,G. (2012) Haplotype-based variant detection from short-read sequencing. arXiv:1207.3907v2 [q-bio.GN].

[9] Rimmer,A., Phan,H., Mathieson,I., Iqbal,Z., Twigg,S.R.F., Consortium,W., Wilkie,A.O.M., McVean,G., and Lunter,G. (2014) Integrating mapping-, assembly- and haplotype-based approaches for calling variants in clinical sequencing applications. *Nature Genetics,* **46**(8), 912–918.

[10] Tan,A., Abecasis,G.R., and Kang,H.M. (2015) Unified representation of genetic variants. *Bioinformatics,* **31**(13), 2202–2204.
